# Successful Conservative Treatment of a Cesarean Scar Pregnancy with Systemically Administered Methotrexate and Subsequent Dilatation and Curettage: A Case Report

**DOI:** 10.1155/2012/248564

**Published:** 2012-01-31

**Authors:** Anis Fadhlaoui, Mohamed Khrouf, Khaled Khémiri, Kais Nouira, Anis Chaker, Fethi Zhioua

**Affiliations:** ^1^Department of Obstetrics and Gynecology, Aziza Othmana University Hospital (Medical University of Tunis), Place du Gouvernement, La Kasba, 1008 Tunis, Tunisia; ^2^Department of Radiology, Aziza Othmana University Hospital (Medical University of Tunis), Place du Gouvernement, La Kasba, 1008 Tunis, Tunisia

## Abstract

Cesarean scar pregnancy is a rare type of ectopic pregnancy associated with severe complications such as uterine rupture, uncontrollable bleeding which may lead to hysterectomy, and definitive infertility. Many therapeutic options are available such as Dilatation & Curetage, excision of trophoblastic tissues using either laparotomy or laparoscopy, systemically administered Methotrexate, and more recently uterine artery embolization. The use of Methotrexate sometimes required laparotomy later because of severe hemorrhage. Through this paper, we demonstrated that viable cesarean scar pregnancy can be managed safely by systemically delivered Methotrexate at the cost of a prolonged followup.

## 1. Introduction

Cesarean scar pregnancy (CSP) is an ectopic pregnancy implanted in the myometrium at the site of a previous cesarean section scar. It is the rarest kind of ectopic pregnancy and may lead to severe complications, such as uterine disruption and severe hemorrhage [1]. Thus, it is important that early and accurate diagnosis is obtained in order to avoid complications and preserve fertility. *Several types of conservative treatment have been used: dilatation and curettage, excision of trophoblastic tissues (laparotomy or laparoscopy) *[2, 3]*, local and/or systemic administration of methotrexate *[4],* bilateral hypogastric artery ligation associated with trophoblastic evacuation, and selective uterine artery embolization combined with curettage and/or MTX administration *[5, 6]. 

In this paper, we describe a case of viable cesarean scar pregnancy that was treated successfully by systemically administered MTX followed by a dilatation and curettage under ultrasound guidance.

## 2. The Case

**Figure 1:**
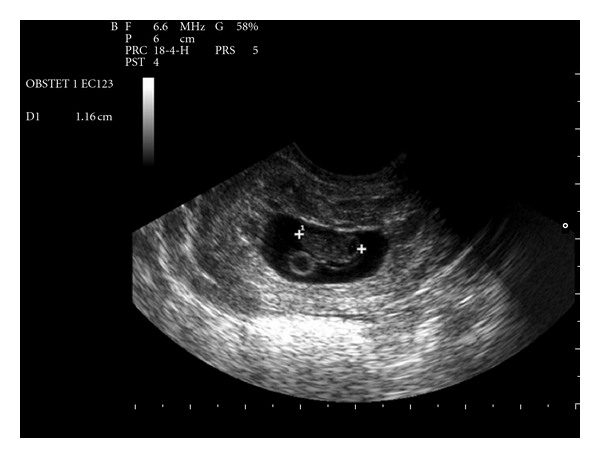
Transvaginal ultrasound image of the scar pregnancy crown-rump length 11.6 mm with fetal cardiac activity.

**Figure 2:**
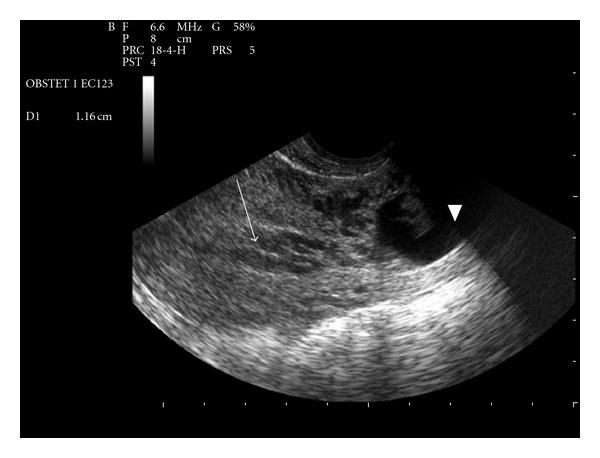
Transvaginal ultrasound image of the scar pregnancy. Gestational sac in the lower anterior wall of the uterus (arrow head), empty uterus (arrow).

**Figure 3:**
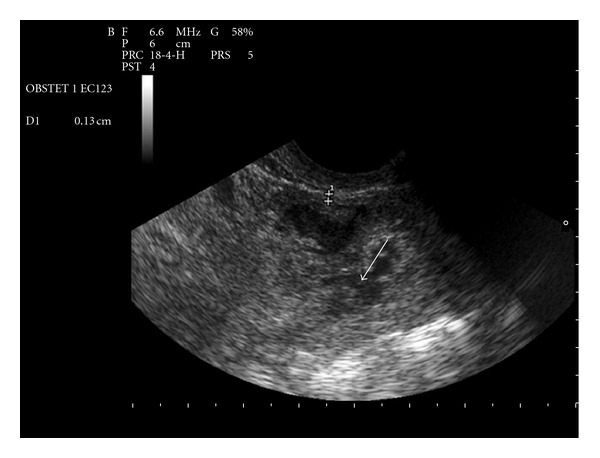
Transvaginal ultrasound image of the scar pregnancy only 1.3 mm of myometrium visualized in the anterior wall of the cervix, empty cervical canal (arrow).

**Figure 4:**
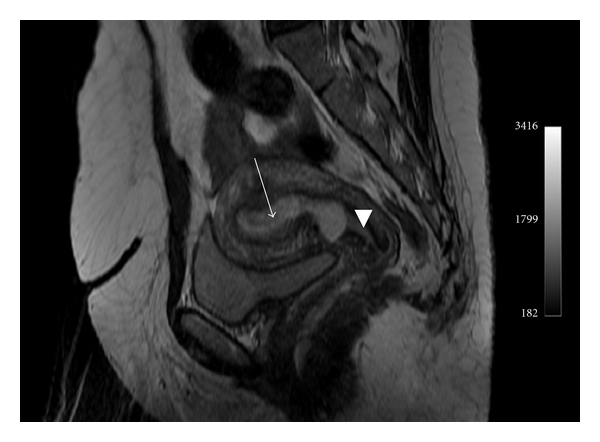
Magnetic resonance imaging cesarean scar pregnancy, empty uterus (arrow), empty cervical canal (arrow head).

**Figure 5:**
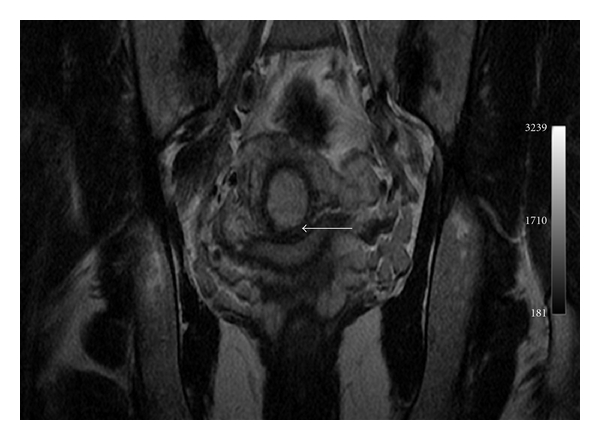
Magnetic resonance imaging gestational sac surrounded by myometrium (arrow head), absence of myometrium between the gestational sac and the bladder (arrow).

A 35-year-old female, gravida 2 para 1, with a previous history of cesarean section, was admitted to hospital for vaginal bleeding at 6-week gestation. Physical examination demonstrated stable vital signs while bimanual examination revealed an enlarged uterus with no adnexal masses. Transvaginal ultrasound revealed a 36 mm well-defined gestational sac with a crown-rump length of 11,6 mm and a fetal cardiac activity in the lower anterior wall of the uterus (Figures 1 and 2). Only 1,3 mm of myometrium was visualized in the anterior wall of the cervix (Figure 3). There was no fluid in the cul-de-sac. The serum level of the ß-subunit of human chorionic gonadotrophin (ß-hCG) was 8332 mUI/mL. These findings were compatible with a cesarean scar pregnancy. A magnetic resonance imaging revealed that the gestational sac was implanted at the site of the previous cesarean section scar, coming down to the serosa without the interposition of the myometrium (Figures 4 and 5), confirming the diagnosis. The patient was counseled regarding her management options, and since she had a prior cesarean in the past, had opted for medical treatment. Thus, she received a first dose (Day 0) of Methotrexate (75 mg: 1 mg/Kg of body weight) intramuscularly. The serum level of the ß-subunit of human Chorionic Gonadotrophin at the 4th day was 18157 mUI/mL requiring a second dose of methotrexate. This second dose was followed by a mild vaginal bleeding. The ß-hCG levels at day 8 decreased to 12562 mUI/mL, and the patient was discharged and followed up by outpatient until a total negativation of ß-hCG levels, at day 34. The transvaginal ultrasound revealed the persistence of a gestational sac without fetus, so we decided to proceed to a D and C under ultrasound guidance. Ten days after the D and C, it was confirmed that the patient had no vaginal bleeding, no pain, and undetectable level of serum ß-hCG. An oral contraception has been prescribed. A control hysterosalpingography was realized, 2 months after the curettage in order to evaluate the scar. It did not reveal any continuity solution nor any fistula.

## 3. Discussion

The increasing rate of cesarean sections in the two last decades has brought into light a set of complications that were not so frequent in the past, including Cesarean scar pregnancy. This condition is defined as a gestation completely surrounded by myometrium and fibrous tissues of the cesarean section scar and separated from endometrium cavity and endocervical canal [7]. The first case was reported in 1978 (Larsen and Solomon) as a postabortal haemorrhage due to what the authors called a uterine scar sacculus [8]. Since then, cases have been reported leading to a better understanding.

The possible incidence of this type of ectopic pregnancy ranges from 1/1800 to 1/2200 pregnancies [9]. The case reported occurred within a period of 12 months during which a total of 62 ectopic pregnancies were diagnosed in our departement.

The pathophysiology of cesarean scar pregnancy remains to be established, but it is possible that the conceptus penetrates the myometrium through a microscopic dehiscent tract of the cesarean scar [3] or the gestational sac implantation occurs in a poor healed cesarean section scar. It may also result from a defect in the endometrium caused by trauma created by procedures in assisted reproduction techniques [10].

The natural history of this condition remains unclear, it may result in a pregnancy that looses its vascular connections while growing, thus causing a spontaneous abortion, or it may continue to grow gaining new stronger vascular connections ending into a low-lying adherent placenta with or without invasion of surrounding organs [11]. Early diagnosis is thus important to avoid serious complications.

The most common symptom is painless vaginal bleeding that may be massive. Since there is no specific clinical sign of the CSP, endovaginal ultrasonography and color flow Doppler are essential for diagnosis. The sonographic criteria for diagnosis [12, 13] are

- empty uterus and empty cervical canal;
- development of the sac in the anterior wall of the isthmic portion;
- a discontinuity on the anterior wall of the uterus demonstrated on a sagittal plane of the uterus running through the amniotic sac;
- absent or diminished healthy myometrium between the bladder and the sac;
- high velocity with low impedance peri-trophoblastic vascular flow clearly surrounding the sac is proposed in Doppler examination.

Miscarriages (Abortion and missed abortion) and cervicoisthmical pregnancies can be sources of confusion in the diagnosis of CSP. Ultrasonography is a precious diagnostic instrument to differentiate these conditions. The differentiating points between CSP and cervicoisthmical pregnancy include the absence of healthy uterine tissues between the sac and the bladder [12].

Because of the rarity of the CSP, there are no optimal lines for therapy. Treatment modalities are either medical or surgical and are sometimes combined. The surgical approach includes radical and conservative procedures. The radical procedure consists in hysterectomy when the uterus is ruptured or if bleeding is uncontrollable. The conservative procedure includes (i) evacuation of the pregnancy and repair of the uterine defect by laparotomy or laparoscopy [12, 14], (ii) dilatation and curettage and excision of trophoblastic tissues using laparotomy or laparoscopy [2, 3, 15], and (iii) bilateral hypogastric artery ligation associated with D and C under laparoscopic guidance [16]. The medical treatment consists of MTX administration locally or systemically [13, 17] some authors combine MTX injected into the sac and potassium chloride injected locally into the fetal heart [18]. The medical treatment requires a prolonged followup (the hCG level takes up to 4 months to return to normal) [19] and implies a high cost. Bleeding may occur following the MTX injection as in the reported case, which may require surgical intervention. Failure of pregnancy resorption and persistance of a relatively large gestational sac may imply a dilatation and curettage or a laparoscopic intervention. Another important issue is the condition of the uterine scar left after medical treatment and its subsequent behavior in future pregnancies (dehiscences are reported) [20].

Another treatment possibility is the uterine artery embolisation UAE [5, 7, 14], which is widely accepted as a conservative treatment in postpartum hemorrhage, in uterine fibroids, it is also considered as the best method to prevent massive bleeding during D and C for cervical pregnancy. Although UAE seems to be promising in treating stable cases, it's not recommended as a primary line therapy.

In our case, since the patient was stable and did not want to have a surgical procedure and since there was no facilities for UAE, we opted for medical treatment. The use of D and C was dictated by the persistence of the gestational sac, despite the negativity of hCG.

The immediate complications of CSP are uterine rupture, severe hemorrhage, need for hysterectomy, and maternal morbidity. Long-term outcomes to be considered after medical, UAE, or conservative surgical treatments are future fertility and recurrence of CSP. A study of 2007 [18] reported a favorable reproductive outcomes and a low recurrence rate.

## 4. Conclusion

In this observation, we demonstrated that viable CSP can be treated safely by systemic methotrexate injection and subsequent dilatation and curettage. Decisions on treatment options should be dictated in part by gestational age, hCG levels, the presence of fetal cardiac activity, the desire of future fertility, and the experience and facilities available. Counselling patients with CSP is not easy, since there is no data about the optimum treatment. More reports are needed to rationalize the treatment modalities on this condition.
